# SARS-CoV-2 and Oral Manifestation: An Observational, Human Study

**DOI:** 10.3390/jcm9103218

**Published:** 2020-10-07

**Authors:** Bruna Sinjari, Damiano D’Ardes, Manlio Santilli, Imena Rexhepi, Gianmaria D’Addazio, Piero Di Carlo, Piero Chiacchiaretta, Sergio Caputi, Francesco Cipollone

**Affiliations:** 1 Department of Medical, Oral and Biotechnological Sciences, University “G. d’Annunzio” Chieti-Pescara, 66100 Chieti, Italy; manlio.santilli@alumni.unich.it (M.S.); imena.rexhepi@gmail.com (I.R.); gianmariad@gmail.com (G.D.); scaputi@unich.it (S.C.); 2 Electron Microscopy Laboratory, University “G. d’Annunzio” Chieti-Pescara, 66100 Chieti, Italy; 3 Clinica Medica Institute, Department of Medicine and Aging Sciences, University “Gabriele d’Annunzio”, Chieti-Pescara, 66100 Chieti, Italy; damianomatrix89@msn.com (D.D.); francesco.cipollone@unich.it (F.C.); 4 CAST, Center of Advanced studies and Technologies, University “G. d’Annunzio” Chieti-Pescara, 66100 Chieti, Italy; piero.dicarlo@unich.it; 5 Department of Psychological, Health & Territorial Sciences, University “G. d’Annunzio” Chieti-Pescara, 66100 Chieti, Italy; 6 Department of Neuroscience, Imaging and Clinical Sciences, University “G. d’Annunzio” Chieti-Pescara, 66100 Chieti, Italy; piero.chiacchiaretta@gmail.com

**Keywords:** SARS-CoV-2, COVID-19, oral manifestation, xerostomia, dysgeusia

## Abstract

The correlation between SARS-CoV-2 and oral manifestations is still controversial. The aim of this observational study was to determine the oral manifestation of the hospitalized patients for COVID-19. A total of 20 patients met the inclusion criteria and gave their signed informed consent. A questionnaire of 32 questions regarding the oral and systemic health condition was administrated to these patients during the convalescence. A descriptive statistic was performed. Data were analysed through the use of χ^2^ test, to assess the statistical significance. A statistically significant increase of about 30% of reporting xerostomia during hospitalization was observed (*p* = 0.02). Meanwhile, a decrease of oral hygiene was observed during the hospitalization, even if a non-statistically significant difference was shown between the two study time points (before and after hospitalization). During the hospitalization period, 25% of patients reported impaired taste, 15% burning sensation, and 20% difficulty in swallowing. An interesting result was that among the systemic conditions, hypertension was observed in 39% of patients and mostly in female patients (62.5%). Further studies are necessary to better understand the symptoms of this new virus in order to faster detect its presence in humans. Probably, a multidisciplinary team following the COVID-19 patients could be of key importance in treating this disease.

## 1. Introduction

The SARS-CoV-2 (Severe Acute Respiratory Syndrome-CoronaVirus 2) is the seventh coronavirus known to infect humans [1,2]. Specifically, it belongs to the family of *Coronaviridae*, of the order *Nidovirales*, comprising large, single, plus-stranded RNA as their genome [3,4]. The new coronavirus SARS-CoV-2 has, like other coronaviruses, with high probability, a zoonotic origin [5]. Among these, α-CoV and β-CoV tend to infect the respiratory, gastrointestinal, and central nervous systems [6]. By studying nucleotide sequences thoroughly, SARS-CoV-2 has been seen to be part of β-CoV with a 79% similarity to the SARS-CoV virus already described in the past decades [7].

The main transmission routes described are direct, as caused by coughing, sneezing, droplets of saliva expelled during the phonation, or indirect by contact with the main body mucous membranes such as oral, ocular, and nasal [8,9,10,11]. Public awareness of the spread of microorganisms and infectious diseases in the dental office among the dentist, auxiliaries, and laboratory personnel has increased significantly [12]. Therefore, several scientific dental societies have produced recommendations on dental activity, specifically for the management of acute dental infections [13]. A recent paper demonstrated, through a survey, that during lockdown period endodontic urgency resulted predominant [14], thus increasing the probability of being infected if measures are not respected through the high aerosol generation during the dental procedures. All over the world, the evolution of the disease diffusion, which today counts high numbers, is being monitored. Specifically, to date, the numbers of infected people still result in a constant increase. The numbers registered by the Center for Systems Science and Engineering (CSSE) at Johns Hopkins University are 33,082,994 infected worldwide and 997,799 deaths (updated to 28 September 2020). On the other hand, cases of recovered patients are also increasing all over the world [7]. These high numbers justify the declared state of pandemic and are undoubtedly attributable to the ease of human infection of the virus itself.

The main symptoms of COVID-19 are fever, tiredness, and dry cough. Some patients may experience soreness and muscle pain, nasal congestion, runny nose, sore throat, or diarrhea, but in severe cases, the infection can cause pneumonia, severe acute respiratory syndrome, kidney failure, and even death [8]. Moreover, the possible asymptomaticity in infected patients is very important and should absolutely not be underestimated [9].

Although there are many studies in the literature on clinical signs in positive SARS-CoV-2 patients, the majority of them have not verified the oral health status of the patients [15].

Possible oral-related symptoms include: hypogeusia, xerostomia, and chemosensory alterations [16]. In fact, xerostomia has been found mainly among COVID-19 patients, due to the neuroinvasive and neurotropic potential of SARS-CoV-2. It was reported that angiotensin-converting enzyme 2 (ACE2)-positive epithelial cells of the salivary gland are an early target of SARS-CoV-2 in rhesus macaques, and these findings suggest that oral manifestations may appear due to impediment of salivary flow in COVID-19-affected patients [17]. In fact, a cross-sectional survey of 108 patients with confirmed SARS-CoV-2 in China observed that 46% of them reported dry mouth, among other symptoms [17,18].

In literature, several cases of oral manifestations apparently related to SARS-CoV-2 have been described [19,20,21]. The importance of good oral hygiene could be an interesting aspect to evaluate a hypothetical relationship between SARS-CoV-2 and oral manifestations. Badran et al. hypothesized that periodontal pocket could be a reservoir for this virus [22]. Periodontopathic bacteria, involved in several process like inflammation, bacteraemia, pneumonia, are also present in the metagenome of positive SARS-CoV-2 patients [23]. Several authors described case reports of SARS-CoV-2-positive patients with oral manifestations potentially compatible with this type of coronavirus [19,20,21]. Moreover, a key factor in the damage of the respiratory system and other organs could be related to the distribution of ACE2 receptors in the human system [24]. Therefore, cells with ACE2 receptor distribution may become host cells for the virus and further cause inflammatory reactions in related organs and tissues, such as the tongue mucosa and salivary glands [25]. It has also been demonstrated that COVID-19 acute infection, along with associated therapeutic measures, could probably contribute to adverse outcomes concerning oral health. In fact, Dziedzic and Wojtyczka, in 2020, showed that it can lead to various opportunistic fungal infections, unspecific oral ulcerations, recurrent oral herpes simplex virus (HSV-1) infection, dysgeusia, fixed drug eruptions, xerostomia linked to decreased salivary flow, ulcerations and gingivitis as a result of the impaired immune system and/or susceptible oral mucosa [26].

It is not clear if the abovementioned manifestations derive from the viral infection, or they could be caused by some systemic deteriorations, based on potential negative reactions to treatments or even possible opportunistic infections [27]. Furthermore, some reports affirm that the oral cavity represents the main channel for infection, considering also several consequences for the dental practice and the role of saliva in identifying COVID-19 [24,25]. One of the latest studies links a higher risk of getting COVID-19 to hyposalivation as well as to taste loss [28].

Although, despite the probable relationship between oral cavity and SARS-CoV-2, to date, there are also many variables that could influence the presence of the oral manifestations. In fact, most patients take a large number of drugs that may produce the oral manifestations, thus the need in evaluating in an observational study the oral manifestation of COVID-19 hospitalized patients.

Based on the hypothesis that oral manifestations could be an initial pattern typical of this virus, the aim of this study was to better understand the relationship between these manifestations and SARS-CoV-2.

## 2. Experimental Section

### 2.1. Study Design and Sample Selection

A total of 20 patients were enrolled in this observational study conducted in a period of one month (from May 2020 to June 2020). The survey was completed by 20 patients who met, during the described period, the inclusion and exclusion criteria. The average age of the participants was 69.2 years. Of these, 55% were male (aged between 44 and 91 years) and 45% female (aged between 35 and 85 years).

A specific anamnestic questionnaire of 32 questions (Appendix A) was submitted to these patients affected by SARS-CoV-2 and hospitalized in “Policlinico ‘SS. Annunziata’ - Chieti, Italy” with the aim to collect information related to health status, oral hygiene habits, and symptoms in the oral cavity before and during the disease manifestation. In addition, a series of questions were also addressed to the Unit of Internal Medicine of the hospital to better know the clinical condition of these patients. This observational study was administered through a printing questionnaire and, prior to completion, the patients gave their informed consent signed to the doctor working in the Unit of Internal Medicine at the “Policlinico ‘SS. Annunziata’” hospital. The patients were free to participate or not (based in a volunteer way) in this observational study. The inclusion criteria were patients of both sex and of any age hospitalized for COVID-19 at the abovementioned hospital able to give their consent to participate in the study. The exclusion criteria were patients of both sex and any age hospitalized for COVID-19 at the Internal Medicine department of the SS Annunziata hospital in Chieti in need of intensive care and/or who were unable to give their consent to participate in the study or who were unable to intend or to want. The methodology adopted for the creation of the questionnaires allowed us to use both quantitative and qualitative variables, differently distributed. All the questionnaires were given to the patients during the doctor routine visits in that department. Then, all the papers were collected in a separate box with all the recommendations to reduce the contagion. The data were analysed after a period of rest from their collection.

### 2.2. Ethical Consideration

The study protocol was approved by the Ethical Committee of the “G. Annunzio University” of Chieti and Pescara: No. 1687 of 22 April 2020. Participants provided their informed consent in accordance with the EU General Data Protection Regulation GDPR (UE) n. 2016/679 and following the Declaration of Helsinki before beginning the completion of the questionnaire. Data collection took place in the time period from 8 May to 1 June 2020.

### 2.3. Statistical Analysis

Some of the answers were codified as dichotomous variables, namely as Yes/No responses, or in general as categorical variables, when a multiple-choice selection was requested. Given the nature of our survey we computed descriptive statistics for most of the questions. For each question, we computed the percentage of the respondents that gave a particular answer with respect to the number of total responses to the question. Answers obtained prior and during the disease manifestation were compared through the use of χ^2^ test, to assess the statistical significance. All statistical comparisons were conducted with a significance level of *p* < 0.05. Statistical analyses were performed using the GraphPad version 8 (GraphPad Software 2365 Northsides, Dr. Suite 560 San Diego, CA, USA) statistical software.

## 3. Results

**Figure 1:**
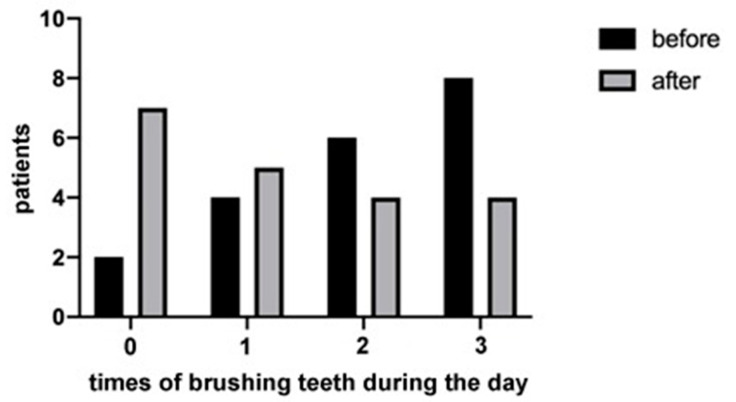
The graphic represents the times the patients used to brush their teeth before and after hospitalization. No statistical significance (*p* = 0.20) was shown between these two time points of the study.

**Figure 2:**
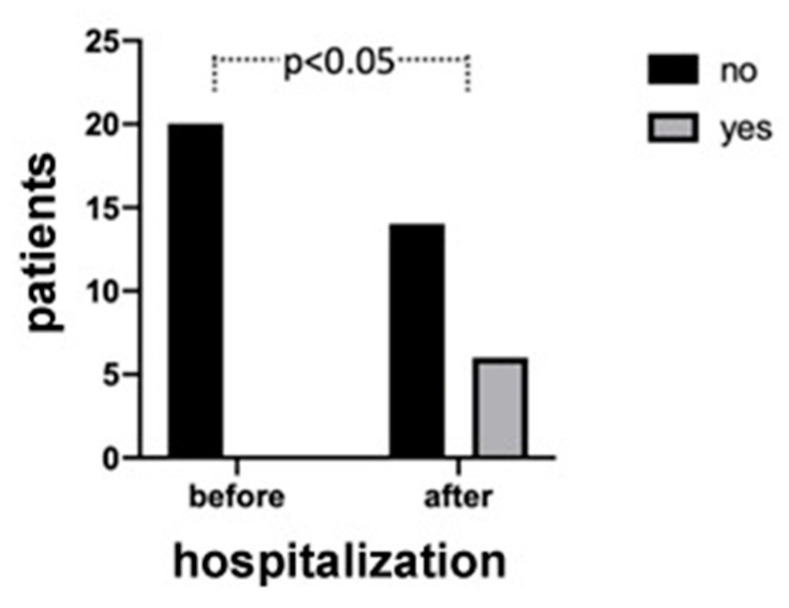
The xerostomia manifestation before and after hospitalization of the patients. A statistical difference was shown between the study time points (*p* = 0.02).

**Figure 3:**
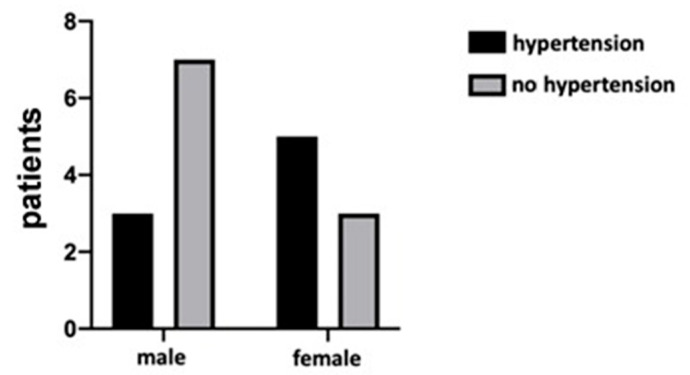
The graph shows the presence of hypertension between males and females. No statistical significance (*p* = 0.34) was shown between the sexes on the presence of hypertension.

The results demonstrated that most of the patients (65%) had more than 20 teeth and used to go to the dentist routinely. Moreover, the majority of participants (90%) were nonsmokers. The 40% of them reported that they brushed their teeth three times a day, before hospitalization, but most of them (70%) did not use dental floss. The patients also reported that during the hospitalization period, the attention to oral hygiene decreased. In fact, the number of patients who did not brush their teeth at all increased during the hospitalization, and the number of those who regularly brush three times a day decreased, as shown in Figure 1. However, the difference between the groups was not statistically significant (*p* = 0.20). Regarding the presence of oral manifestations (i.e., xerostomia), none of the patients reported xerostomia before contracting the virus, whilst during hospitalization the percentage increased to 30%. The difference between the two study time points was statistically significant (*p* = 0.02), as shown in Figure 2. In addition, during the hospitalization period, 25% of patients reported impaired taste, 15% burning sensation, and 20% difficulty in swallowing. Finally, by comparing these data and the onset of some manifestations between sex and age, no statistically significant results emerged, although a trend in some of these was detectable (please see Appendix B). Among the latter’s, the presence of hypertension was found in 40% of patients, mostly in female patients (62.5%), as shown in Figure 3. Furthermore, an interesting data aspect was that the burning sensation of the mouth was present only in female patients. In addition, 15% of patients were affected by diabetes, 15% by obesity, and 25% presented thyroid disorders such as hypothyroidism or hyperthyroidism. Of note, 95% of the patients were given the following drugs: lopinavir/ritonavir and/or hydroxychloroquine, in combination with other specific drugs for the various systemic pathologies they presented.

## 4. Discussion

The aim of this observational study was to better understand the relationship between SARS-CoV-2 and oral manifestations before and during the hospitalization. Several clinicians have observed many extrapulmonary manifestations of COVID-19. In fact, the recent literature suggests that the hematologic, cardiovascular, renal, gastrointestinal and hepatobiliary, endocrinologic, neurologic, ophthalmologic, and dermatologic systems can all be implicated [29,30]. On the other hand, numerous studies have drawn attention to the oral cavity as the main route of infection [28].

Although recent evidence suggests a relevant role of the oral cavity and its mucosae in the transmission and in the pathogenicity of SARS-CoV-2, as the entrance to the body of the virus, its protective or aggravating element for the infection and progression of the virus is still controversial [28]. It has been demonstrated that there is an association between periodontitis and a higher risk of increased gravity of COVID-19 in periodontopathic patients [31]. Most of the individuals (65%) in our sample had more than 20 teeth and they used to go to the dentist for control visits, demonstrating the importance given to the oral health condition. Moreover, about 14 out of 20 of the patients with COVID-19 diagnosis had performed extractions due to periodontitis.

In addition, regarding the presence of xerostomia, only 30% of the patients developed this symptom during the period of hospitalization. These data are relevant because xerostomia has also been found in a relatively high proportion of COVID-19 patients from Chinese researchers [32]. On the other hand, these results should be carefully discussed. In fact, it has been shown that xerostomia can also be induced by different drug therapies such as: antidepressants, antipsychotics, anticholinergics, antihypertensives, antihistamines, and sedatives [33]. Furthermore, there is strong evidence that xerostomia is very common in diabetic patients and may be present in >50% of cases, and recently it was reported that the use of artificial saliva spray was shown to be effective in the treatment of xerostomia in type 1 and type 2 diabetes [34,35]. However, in our study, only 15% of patients were affected by diabetes (not specified if type 1 or type 2).

In fact, 56% of the patients enrolled in our study had these kinds of therapies, but only 5% of them manifested xerostomia during hospitalization. These data deserve attention, because the symptom of xerostomia was manifested by patients affected by COVID-19 and enrolled in our study in 30% of cases, regardless of the drug therapy followed prior to admission. Therefore, this oral manifestation can probably be linked to the disease itself. Indeed, it has been demonstrated that the salivary glands are a reservoir of the virus, thus the contagion of people by way of saliva droplets [36].

Interestingly, the SARS-CoV-2 infection has been shown to be more severe in individuals over 50 years old and with the presence of associated comorbidities such as diabetes, cardiovascular problems, and diseases involving the nervous system. These disorders have been associated with hyposalivation; in our case 15% of patients were affected by diabetes, 15% by obesity, 39% by hypertension, and 25% presented thyroid disorders such as hypothyroidism or hyperthyroidism, but none of them, before being hospitalized, reported having xerostomia. Therefore, the onset of this symptom can be associated with the drug therapy administered for the treatment of COVID-19 and also with the infectious and inflammatory processes activated by the virus itself. Regarding other symptoms such as altered taste, 25% of the participants said they had dysgeusia. This is a very important finding and in line with recent publications on this topic, which attest to 33% the frequency of COVID-19 patients who report having this symptom [37]. Indeed, dysgeusia can be described as one of the early symptoms of COVID-19 infection. Clinically, these data may allow easier identification of pre-symptomatic or asymptomatic patients. Moreover, the diagnosis of this oral manifestation may significantly reduce disease transmission, especially when diagnostic tests are not readily available and/or unpredictable [38].

Focusing on the patient’s systemic conditions, it appears significant that most of the patients hospitalized for COVID-19 had previous systemic conditions such as hypertension, heart disease, oncological pathologies, pathologies affecting the thyroid gland, diabetes, and pathologies affecting the respiratory system. Furthermore, only one patient in his medical history did not report any previous pathology. In addition, it should be noted that, in a recent study on 5700 patients, the most common comorbidities were hypertension in 56.6% of cases, obesity in 41.7%, and diabetes in 33.8% of patients with diagnosis of COVID-19 [39]. Our results are in agreement with these data. In fact, about 39% of our patients had hypertension. It is almost known that such pathologies are aggravated by factors such as smoking. An interesting result that emerged from our study is that approximately 90% of the participants were nonsmokers. In the literature, there are several studies that analysed the relationship between COVID-19 and smoking. According to the World Health Organization (WHO), no studies examined tobacco use and the risk of infection or the risk of hospitalization with COVID-19 among smokers [40]. In fact, the majority of the studies in the literature are observational reports, and they reported the prevalence of smoking amongst hospitalized COVID-19 patients [40].

As for the presence of cardiovascular diseases, the results of our study show that 50% of the participants had cardiovascular diseases, specifically 78% of them suffer from hypertension. Currently, the literature is controversial, also in the management of patients with hypertension since the SARS-CoV-2 uses ACE2 as a cell entry receptor [41]. It is unclear whether uncontrolled blood pressure is a risk factor for acquiring COVID-19, or whether controlled blood pressure among patients with hypertension is or is not less of a risk factor [42].

Although this observational study reports interesting data of 20 COVID-19 hospitalized patients, it has different limitations. Firstly, the small sample size, only 20 patients enrolled. This was given from different limitations on performing the study during the pandemic; the difficulty in enrolling patients with the abovementioned criteria during that period and the difficulty in having personnel available to administrate the questionnaire.

After the results raised, the questionnaires probably should have been done in a more specific way to better understand on which day of the disease the symptoms appear and if they had prior to the first symptom the symptoms they reported.

## 5. Conclusions

This study demonstrates the importance of the close link between SARS-CoV-2 and oral manifestations. There is no scientific evidence in the literature that certifies which oral symptoms SARS-CoV-2 can actually cause. In fact, from the analysis of our data, it is hard to notice that clinical conditions that patients manifest are due to the SARS-CoV-2. The presence of xerostomia in our patients suggests a symptom given by the virus, but it must always be correlated with the patient’s therapy. In addition, it may be essential to carry out the measurement of the salivary flow before and after the COVID-19 diagnosis to demonstrate a close correlation of it with the virus. Furthermore, the dysgeusia present in only 25% of our study suggests that this symptom may be a warning signal for the patients. Finally, the reduction of oral hygiene conditions in the hospitalized patient (even if it was not the focus of this study) suggests how important it is to have a team specialized in dentistry within hospitals.

Further studies are necessary to better understand the symptoms of this new virus in order to faster detect its presence in humans; probably, a multidisciplinary team following the COVID-19 patients could be of key importance.

## Figures and Tables

**Figure A1:**
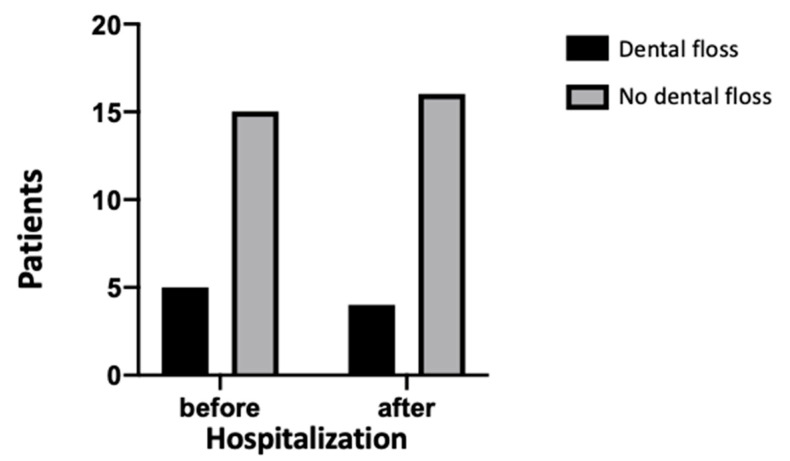
Use of dental floss before and after hospitalization of the patients. No statistical difference was shown between the study time points (*p* > 0.99).

**Figure A2:**
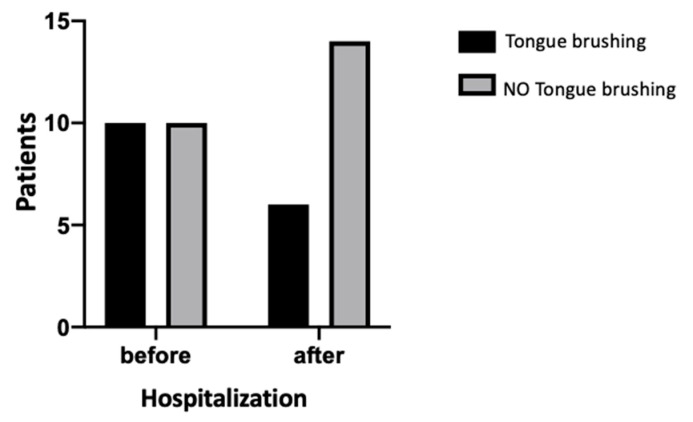
Habit of tongue-brushing before and after hospitalization of the patients. No statistical difference was shown between the study time points (*p* = 0.333).

**Figure A3:**
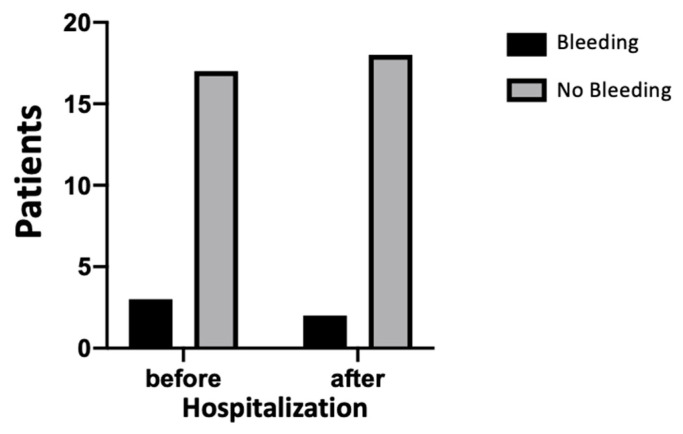
Bleeding of gums before and after hospitalization of the patients. No statistical difference was shown between the study time points (*p* > 0.99).

**Figure A4:**
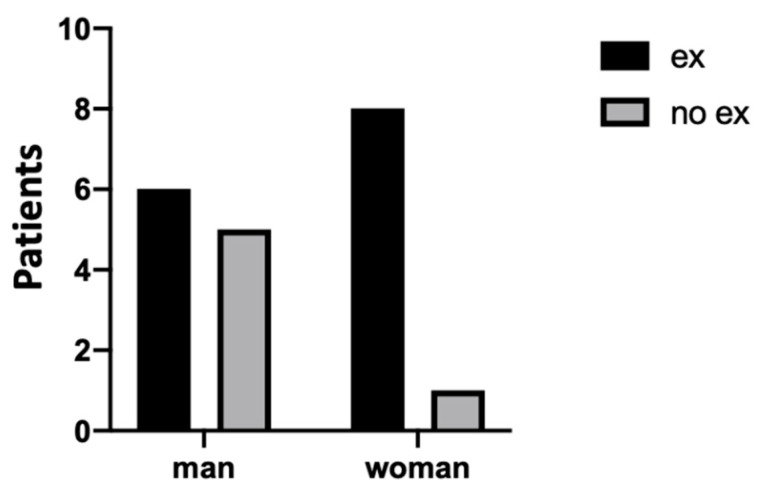
Tooth extraction (EX) carried out between males and females. No statistical significance (*p* = 0.15) was shown between the sexes on the tooth extraction performed.

**Figure A5:**
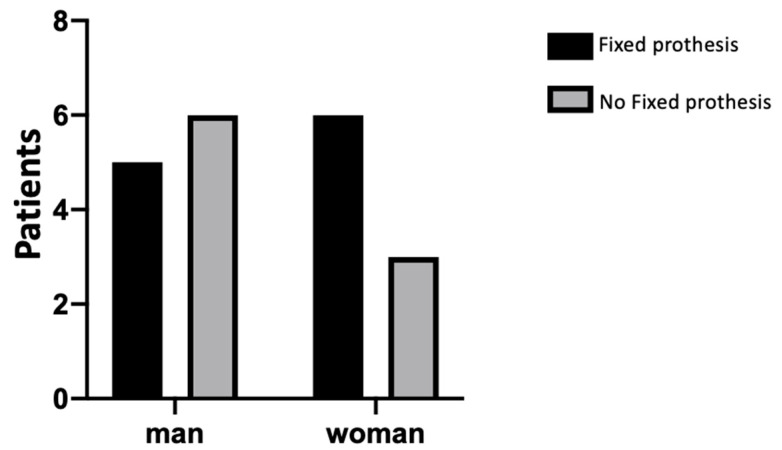
Difference between fixed-prosthesis wearers between males and females. No statistical significance (*p* = 0.40) was shown between the sexes.

**Figure A6:**
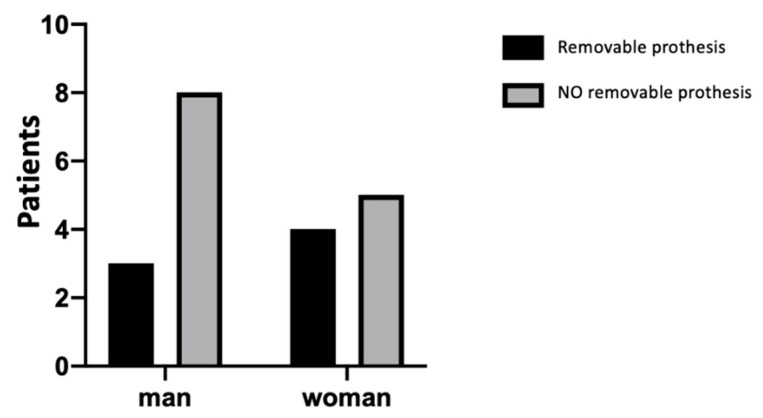
Difference between removable-prosthesis wearers between males and females. No statistical significance (*p* = 0.64) was shown between the sexes.

**Figure A7:**
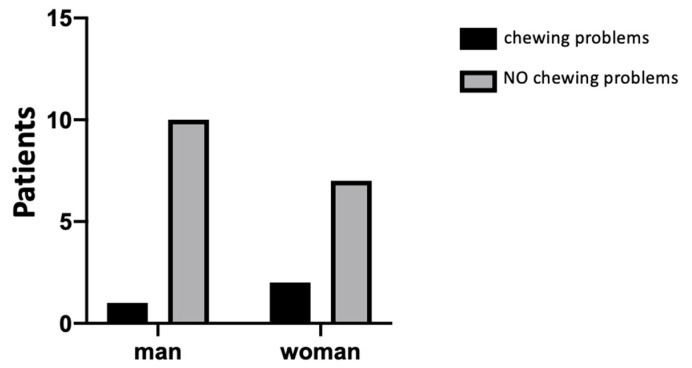
Difference between chewing problems between males and females. No statistical significance (*p* = 0.56) was shown between the sexes.

**Figure A8:**
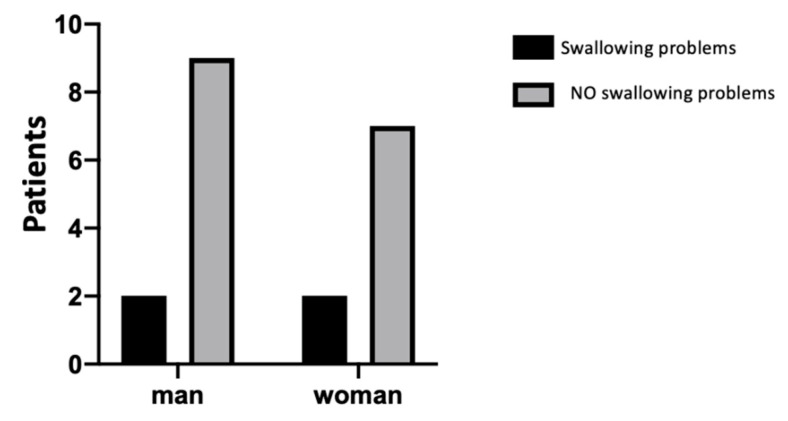
Difference between swallowing problems between males and females. No statistical significance (*p* > 0.99) was shown between the sexes.

**Figure A9:**
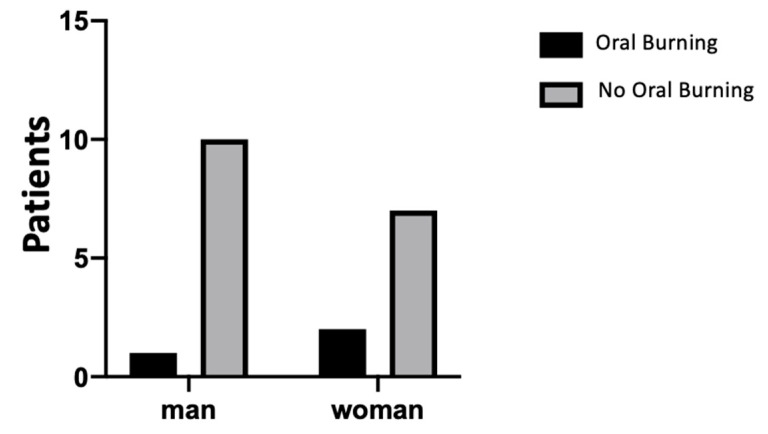
Difference between oral burning sensation between males and females. No statistical significance (*p* = 0.56) was shown between the sexes.

**Figure A10:**
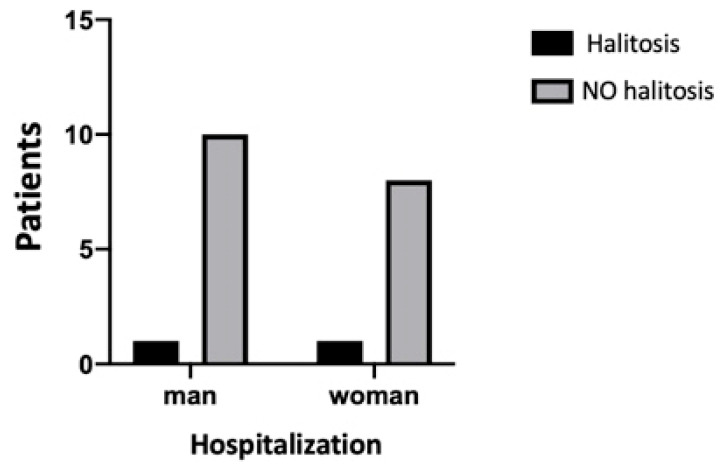
Difference of halitosis perception between males and females. No statistical significance (*p* > 0.99) was shown between the sexes.

**Figure A11:**
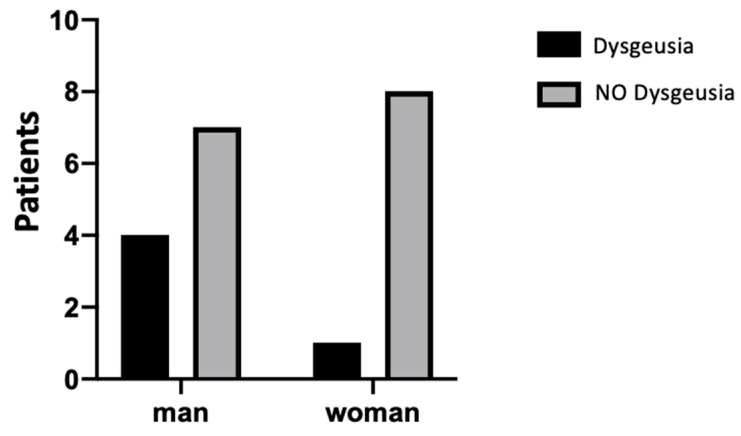
Difference of dysgeusia perception between males and females. No statistical significance (*p* = 0.31) was shown between the sexes.

## Appendix A. Questionnaire

1. Age	
2. Sex	Male
	Female
3. Place of origin	
4. How many teeth do you have in your mouth?	>20 teeth
	10–19 teeth
	1–9 teeth
	totally edentulous patient
5. Are you a smoker?	Yes
	No
6. When did you last go to the dentist?	performed within 6 months
	performed within 1 year
	more than a year since the last visit
7. How many times did you brush your teeth in a day before being hospitalized?	times/after meals daily
	times daily
	1 time daily
	Never
8. Did you use interdental brushes?	Yes
	No
9. If yes, how often weekly?	Yes, but it has been treated
	Yes, but I neglect the problem
	No, I wasn’t told
	I don’t know
10. When brushing your teeth, do your gums bleed?	Yes
	No
11. Did you clean your tongue before hospitalization?	Yes
	No
12. Did the dentist ever tell you that you have gum problems, gum infections or inflammation?	Yes, but it has been treated
	Yes, but I neglect the problem
	No, I wasn’t told
	I don’t know
13. Did the dentist extract your teeth because they had high mobility?	Yes
	No
14. Are you wearing a fixed prosthesis?	Yes
15. Are you wearing a removable prosthesis?	Yes, total
	Yes, partial
	Yes, both total and partial
	No
16. Has anyone in your family of origin (father, mother, siblings, uncles, …) had gum problems such as periodontitis?	Yes
	No
	I don’t know
17. Do you suffer from xerostomia (dry mouth)?	Yes
	No
18. Which home oral hygiene aids do you use now that you are hospitalized?	toothbrush + toothpaste + floss
	prosthesis brush + toothpaste + tablets
	toothbrush + toothpaste
	nothing
19. How many times do you brush your teeth a day, now that you are hospitalized?	1230
20. Do you still clean your tongue now?	Yes
	No
21. Do you currently bleed from your gums while cleaning your teeth?	Yes
	No
22. Did you have any chewing problems during the period of illness (COVID-19)?	Yes
	No
23. Did you have any swallowing problems during the period of illness (COVID-19)?	Yes
	No
24. Did you experience a burning sensation in your mouth during the period of your illness (COVID-19)?	Yes
	No
25. Did you experience halitosis during the period of your illness (COVID-19)?	Yes
	No
26. During the period of your illness (COVID-19) did you have tooth problems/pain?	Yes
	No
27. During the period of the disease (COVID-19) did you have any taste alterations?	Yes
	No
28. Did you suffer from xerostomia during the period of your illness (COVID-19)?	Yes
	No
29. Do you suffer from diabetes?	Yes
	No
30. Do you suffer from cardiovascular disease?	Yes
	No
31. Do you suffer from senile dementia?	Yes
	No
32. Did you experience any other oral problems during hospitalization?	Yes
	No
